# Risk factors for hypertension in patients with type 2 diabetes mellitus in the Casablanca-Settat region, Morocco: a cross-sectional study

**DOI:** 10.11604/pamj.2025.51.38.46334

**Published:** 2025-06-09

**Authors:** Houda El Alami, Meryem Bouqdayr, Khaoula Errafii, Mustapha Lkhider, Najib Al Idrissi, Hassan Ghazal, Lahcen Wakrim, Omar Abidi, Naima Khlil, Abderrahim Naamane, Abderrahmane Maaroufi, Salsabil Hamdi

**Affiliations:** 1 Research and Teaching Department, Virology and Public Health Laboratory, *Institut Pasteur du Maroc*, Casablanca, Morocco,; 2 Laboratory of Biology and Health, Faculty of Sciences Ben M´sick, Hassan II University of Casablanca, Casablanca, Morocco,; 3 African Genome Center, Mohammed VI Polytechnic University, Bengurir, Morocco,; 4 Faculty of Sciences and Techniques of Mohammedia, Hassan II University of Casablanca, Casablanca, Morocco,; 5 Department of Surgery, School of Medicine, Mohammed VI University of Health Sciences, Casablanca, Morocco,; 6 Department of Sports Sciences, Laboratory of Sports Sciences and Performance Optimization, Royal Institute of Executive Management for Youth and Sport, Rabat, Morocco,; 7 Laboratory of Virology Unit, Immunovirology, *Institut Pasteur du Maroc*, Casablanca, Morocco,; 8 Laboratory of Human Molecular Genetics and Medical Genomics, *Institut Supérieur des Professions Infirmières et Techniques de Santé* (ISPITS), Casablanca, Morocco,; 9 Laboratory of Chemistry, Biochemistry, Nutrition and Environment, Faculty of Medicine and Pharmacy, University Hassan II of Casablanca, Casablanca, Morocco,; 10 Institut Pasteur du Maroc, Casablanca, Morocco

**Keywords:** Hypertension, type 2 diabetes mellitus, risk factors, CVD, Morocco

## Abstract

**Introduction:**

hypertension in patients with type 2 diabetes mellitus (T2DM) is a major problem that increases the risk of morbidity and mortality. Although the prevalence of this disease is high in the T2DM population, the risk factors for it remain understudied. The objective of this study is to identify the risk factors combined with hypertension in Moroccan patients with T2DM.

**Methods:**

this cross-sectional study was conducted between January 2017 and July 2018 in primary healthcare centers. A total of 503 T2DM patients were included. Inclusion criteria: adults (≥18 years) with T2DM for ≥1 year. Exclusion criteria: type 1 diabetes, gestational diabetes, cognitive impairment, or missing data. Data on demographics, clinical history, and biochemical markers were analyzed using SPSS. Logistic regression identified independent risk factors, reported as adjusted OR with 95% CI. A p-value <0.05 was considered statistically significant.

**Results:**

a total of 503 patients with T2DM were included (71% female, 29% male), with a mean age of 58.4 ± 10.2 years. The prevalence of hypertension was 70.2%. Patients with hypertension were significantly older (mean age: 61.2 ± 9.8 years vs. 52.6 ± 10.5 years, p ≤ 0.001). The most common cardiovascular risk factors were dyslipidemia (54.1%), obesity (mean BMI: 30.8 ± 5.6 kg/m^2^), and a sedentary lifestyle (50.6%). Multivariable logistic regression identified older age (OR: 4.74, 95% CI: 2.47-9.10; p ≤ 0.001), longer diabetes duration (OR: 2.28, 95% CI: 1.14-4.53; p = 0.018), BMI levels (OR: 1.05, 95% CI: 1.00-1.10; p = 0.019), dyslipidemia (OR: 2.07, 95% CI: 1.28-3.33; p = 0.003), and therapeutic modifications (OR: 1.98, 95% CI: 1.11-3.53; p = 0.020) as significant risk factors.

**Conclusion:**

in this study, we found that hypertension is highly prevalent among patients with T2DM. Our findings indicate that older age, longer diabetes duration, therapeutic modifications, dyslipidemia, and higher BMI levels are significant risk factors for diabetes.

## Introduction

Type 2 diabetes mellitus (T2DM) is a real global health problem. The International Diabetes Federation (IDF) reported that diabetes affects over 537 million adults worldwide. This number is expected to reach 643 million by 2030 and 783 million by 2045 [1]. The World Health Organization (WHO) predicts that diabetes could be the seventh leading cause of death by 2030 [2]. The prevalence of this disease is increasing rapidly due to changes in case determination and diagnostic criteria, but especially due to lifestyle changes in rapidly developing countries. Expanded diagnostic criteria and increased screening have contributed to earlier detection, but the main drivers remain poor diets, sedentary habits, and rising obesity. Urbanization and environmental factors further exacerbate the trend. Addressing this growing burden requires comprehensive public health interventions and widespread lifestyle modifications.

Cardiovascular disease (CVD) is the leading cause of morbidity and mortality in patients with diabetes. This mortality is greatest in low- and middle-income countries and lowest in high-income countries [3]. Individuals with diabetes have a double or greater relative risk of cardiovascular disease versus those without diabetes [4]. In addition, hypertension is considered a major risk factor that increases the risk of long-term vascular complications of T2DM, namely, stroke, chronic kidney disease, heart disease, peripheral vascular disease, and premature death [5-8]. Furthermore, the prevalence of hypertension is up to three times higher in patients with T2DM compared to healthy counterparts [9]. Currently, hypertension appears in 50-80% of patients with T2DM, which constitutes more than 90% of the diabetic population [10,11]. Since hypertension is particularly common in T2DM, it is suggested that insulin resistance could be involved in the pathogenesis of hypertension. On the other hand, a prospective cohort study of 12,550 adults found that patients with hypertension were almost 2.5 times more likely to develop T2DM than those with normal blood pressure [10-12].

Indeed, a Framingham study on individuals with diabetes found that hypertension is a significant risk factor leading to the presence of CVD in this population [13]. The results of this and other studies suggest that patients with both diabetes and hypertension have a doubled risk of cardiovascular events and mortality compared to those with normal blood pressure [9]. Moreover, evidence indicates that the coexistence of diabetes and hypertension not only accelerates CVD progression but also increases the risk of other complications, such as chronic kidney disease and stroke. These findings underscore the importance of early detection and aggressive management of both conditions to reduce overall morbidity and mortality.

However, the prevention of high blood pressure was associated with a decreased risk of T2DM-related complications, including death, stroke, and the need for retinal photocoagulation [14]. This cross-sectional study aimed to determine the prevalence of hypertension among patients with T2DM and their risk factors in Morocco.

## Methods

**Study design and setting:** this was a cross-sectional study conducted between January 2017 and July 2018 in health centers across the Casablanca-Settat region, Morocco. The study included general medicine consultations, hospitals, and the Pasteur Institute of Morocco.

**Study population:** this cross-sectional study included 503 patients with T2DM from primary healthcare centers in the Casablanca-Settat region, Morocco. The sample size was determined using the Cochran formula for prevalence studies, considering a 95% confidence level, an estimated hypertension prevalence of 50% (to maximize variability), and a margin of error of 5%, yielding a minimum required sample of 385 participants. To ensure better representativeness, 503 patients were enrolled. A convenience sampling method was used, recruiting patients attending regular diabetes follow-ups during the study period. While this approach allows for practical recruitment, it may introduce selection bias, which was mitigated by enrolling patients across different days and time slots. Inclusion criteria: adults (≥18 years) with a confirmed diagnosis of T2DM for at least one year. Exclusion criteria: type 1 diabetes, gestational diabetes, cognitive impairment, or incomplete clinical data.

**Data collection:** data was collected using structured questionnaires, medical records, and laboratory tests. The questionnaire included sections on sociodemographic information (age, gender, marital status, education level, income, medical coverage, area of residence, and ethnicity), lifestyle factors (smoking status and physical activity), and clinical characteristics (duration of diabetes, presence of hypertension, dyslipidemia, medication use, and therapeutic modifications). Anthropometric measurements were recorded following standard protocols: body mass index (BMI) was calculated as weight (kg) divided by height squared (m^2^). Blood pressure (BP) was measured using a calibrated sphygmomanometer, with hypertension defined as systolic BP ≥140 mmHg and/or diastolic BP ≥90 mmHg, or the use of antihypertensive medication [15]. Laboratory tests were performed to assess fasting blood glucose (FBG), glycated hemoglobin (HbA1c), lipid profile (total cholesterol, triglycerides, HDL-C, LDL-C). These tests were conducted in certified laboratories using standard biochemical analysis methods. Trained healthcare professionals collected patient information during routine follow-ups, ensuring consistency and accuracy. Each participant provided written informed consent, and all data were anonymized before analysis to maintain confidentiality.

**Definitions:** hypertension was diagnosed based on the European Society of Cardiology (ESC) guidelines [15]. Obesity was defined as a BMI ≥30 kg/m^2^ according to WHO standards [16]. Dyslipidemia was classified as total cholesterol >200 mg/dL, LDL-C >100 mg/dL, HDL-C <40 mg/dL (men) or <50 mg/dL (women), or triglycerides >150 mg/dL [17].

**Data analysis:** statistical analyses were conducted using SPSS software (version 25, IBM Corp., Armonk, NY, USA). Continuous variables were described as mean ± standard deviation (SD) or median (interquartile range) and compared using the independent samples t-test or Mann-Whitney U test, depending on data normality. Categorical variables were presented as percentages and compared using the Chi-square test or Fisher´s exact test, when appropriate [18]. All risk factors associated with hypertension were identified using binary logistic regression analysis. Bivariable logistic regression was initially performed to determine the association between each independent variable and hypertension. Variables with p < 0.5 in the bivariable analysis were included in the multivariable logistic regression model to adjust for potential confounders and mediators. Adjusted odds ratios (OR) with 95% confidence intervals (CI) were reported to identify significant predictors of hypertension, and a p-value < 0.05 was considered statistically significant [19]. Results were interpreted based on adjusted odds ratios with 95% confidence intervals, and only statistically and clinically relevant associations were considered significant. When necessary, sensitivity analyses were performed to confirm the findings.

**Ethical considerations:** this study was conducted in accordance with the ethical principles outlined in the Declaration of Helsinki (2008) and the CIOMS international ethical guidelines (2002). Ethical approval was obtained from the Biomedical Research Ethics Committee of Rabat, Morocco, which issued a favorable opinion for the study. The committee follows established guidelines, including the Directive 2001/20/CE of the European Parliament, Moroccan medical regulations, and sociocultural considerations. The committee is registered with the U.S Department of Health and Human Services Office for Human Research Protections (OHRP) under registration number IORG0006594. All participants provided written informed consent before enrollment. For illiterate participants, a witness of their choice assisted in the consent process. To ensure confidentiality, all data were anonymized and securely stored, with unique identification codes assigned to each participant.

## Results

**General characteristics of the study population:** among the 503 T2DM patients (71% female, 29% male), 70.2% were hypertensive. Hypertensive patients were significantly older (p ≤ 0.001); however, no association was found with gender (p = 0.320). The prevalence of illiteracy was higher among hypertensive patients (62.9% vs. 48.6%, p = 0.053), and widowhood was more frequent (22.9% vs. 13.4%, p = 0.049). The Saharan ethnicity was significantly more prevalent in the hypertensive group (5.8% vs. 1.3%, p = 0.034) (Table 1). Nearly half of hypertensive patients had diabetes for more than 10 years (42.9% vs. 25.3%, p ≤ 0.001). Use of insulin with oral hypoglycemic agents was more common in hypertensive patients (19.4% vs. 10.1%, p = 0.030). Therapeutic modifications were more frequent among hypertensive patients (49.8% vs. 31.7%, p ≤ 0.001), as was treatment for dyslipidemia (54.1% vs. 35.1%, p ≤ 0.001) (Table 2). Hypertensive patients had higher BMI (30.82 vs. 29.06 kg/m^2^, p ≤ 0.001), systolic BP (140 vs. 120 mmHg, p ≤ 0.001), and diastolic BP (80 vs. 70 mmHg, p ≤ 0.001) compared to non-hypertensive patients.

**Table 1:** distribution of sociodemographic parameters according to hypertension

Parameters	Hypertension		p-value
	Yes (N=353)	No (N=150)	
**Gender (%)**			
Female	71	29	0.320
Male	65.3	34.7	
**Age (%)**			
30-49 years	16.8	40.7	≤0.001
50-59 years	31.9	35.9	
≥ 60 years	51.3	23.4	
**Education level (%)**			
Illiterate	62.9	48.6	0.053
Primary	19.5	24.3	
Secondary	8.6	13.5	
Higher secondary	4.9	7.4	
Graduate degree	4.0	6.1	
**Marital status (%)**			
Single	4.9	8.7	0.049
Married	61.7	67.8	
Divorced	10.6	10.1	
Widowed	22.9	13.4	
Consanguinity (%)	16.3	16.9	0.868
**Ethnicity (%)**			
Arab	86.9	87.3	0.034
Berber	7.3	11.3	
Saharan	5.8	1.3	
**Residence (%)**			
Rural	8.8	8.7	0.984
Urban	91.2	91.3	

**Table 2:** distribution of clinical and biological parameters according to hypertension

Parameters	Hypertension		p-value
	Yes (N=353)	No (N=150)	
**Duration of diabetes^1^**			
5 years	22.7	45.3	≤0.001
5-10 years	34.4	29.3	
>10 years	42.9	25.3	
**Diabetes treatment^1^**			
Hygienic rules only	8.3	11.4	0.030
OHA	46.9	56.4	
Insulin only	25.4	22.1	
Insulin + OHA	19.4	10.1	
Therapeutic modification**^1^**	49.8	31.7	≤0.001
Regular medical monitoring**^1^**	84.6	80.8	0.298
Self-monitoring of blood sugar**^1^**	70.4	66	0.332
**Behaviours^1^**			
Sedentary lifestyle	50.6	42.3	0.090
Smoking	7.9	12	0.148
Dyslipidemia**^1^**	54.1	35.3	≤0.001
BMI in Kg/m**^2^ ^2^**	30.82 (27.33-34.96)	29.06 (26.37-32.03)	≤ 0.001
SBP in mmHg**^2^**	140 (130-160)	120 (110-130)	≤ 0.001
DBP in mmHg**^2^**	80 (70-80)	70 (60-80)	≤ 0.001
**Paraclinical findings^2^**			
HbA1C (%)	8.40 (7.00-10.40)	8.40 (7.00-11.00)	0.322
FBG (mg/dl)	181 (135-243)	187 (130-266)	0.423
Total Cholesterol (mg/dl)	191 (164-227)	181 (154-220)	0.229
Triglycerides (mg/l)	131 (91-185)	112 (86-167)	0.056
HDL-C (mg/dl)	50 (42-57)	52 (43-59)	0.344
LDL-C (mg/dl)	110 (94-135)	109 (97-133)	0.975

1 : expressed in frequency (%); ^2^: expressed in median (interquartile range); OHA: oral hypoglycemic agents; SBP: systolic blood pressure; DBP: diastolic blood pressure; FBG: fasting blood glucose; HDL: high-density lipoprotein; LDL: low-density lipoprotein

**Prevalence and correlations of hypertension:** we tested the association between all independent variables and hypertension using binary logistic regression. In the bivariable analysis, significant candidates for the final model included age 50-59 years (OR = 2.15, 95% CI: 1.31-3.51, p = 0.002) and ≥60 years (OR = 5.29, 95% CI: 3.16-8.87, p ≤ 0.001), widowhood (OR = 3.05, 95% CI: 1.27-7.32, p = 0.012), diabetes duration of 5-10 years (OR = 2.33, 95% CI: 1.45-3.75, p ≤ 0.001) or >10 years (OR = 3.37, 95% CI: 2.08-5.46, p ≤ 0.001), treatment with OHAs and insulin (OR = 2.65, 95% CI: 1.17-6.02, p = 0.019), therapeutic modifications (OR = 2.14, 95% CI: 1.41-3.26, p ≤ 0.001), BMI (OR = 1.05, 95% CI: 1.02-1.09, p = 0.002), and hypolipidemia treatment (OR = 2.16, 95% CI: 1.45-3.20, p ≤ 0.001) (Table 3).

**Table 3:** univariable and multivariable analysis of risk factors for developing hypertension in T2DM patients in Morocco

Parameters	Univariable analysis		Multivariable analysis	
	ORs (95% CI)	p-value	Adjusted ORs (95% CI)	p-value
Sex (female vs male)	1.30 (0.77-2.18)	0.321	-	-
**Age, years**				
30-49	References		References	
50-59	2.15 (1.31-3.51)	0.002	2.00 (1.10-3.64)	0.023
≥ 60	5.29 (3.16-8.87)	≤ 0.001	4.74 (2.47-9.10)	≤ 0.001
**Marital status**				
Single	References		References	
Married	1.63 (0.76-3.49)	0.204	1.39 (0.55-3.4)	0.478
Divorced	1.88 (0.73-4.82)	0.185	1.39 (0.44-4.38)	0.568
Widowed	3.05 (1.27-7.32)	0.012	1.27 (0.44-3.67)	0.652
**Education level**				
Illiterate	References		References	
Primary	0.62 (0.38-1.00)	0.054	0.69 (0.37-1.27)	0.239
Secondary	0.49 (0.26-0.92)	0.027	0.75 (0.35-1.60)	0.469
Higher secondary	0.50 (0.22-1.13)	0.099	1.47 (0.50-4.31)	0.474
Graduate degree	0.51 (0.21-1.23)	0.135	0.48 (0.17-1.40)	0.183
**Residence**				
Rural	References			
Urban	1.00 (5.51-1.98)	0.984	-	-
**Ethnicity**				
Arab	References		References	
Berber	0.64 (0.33-1.23)	0.188	0.45 (0.20-1.01)	0.055
Saharan	4.39 (1.01-19.08)	0.048	7.31 (0.88-60.59)	0.065
**Duration of diabetes, years**				
<5	References		References	
5-10	2.33 (1.45-3.75)	≤ 0.001	2.13 (1.17-3.86)	0.013
>10	3.37 (2.08-5.46)	≤ 0.001	2.28 (1.14-4.53)	0.018
**Diabetes treatment**				
Hygienic rules only	References		References	
OHA	1.14 (0.59-2.20)	0.686	0.87 (0.39-1.96)	0.749
Insulin only	1.58 (0.77-3.24)	0.212	0.68 (0.25-1.84)	0.449
Insulin + OHA	2.65 (1.17-6.02)	0.019	0.84 (0.26-2.62)	0.765
No regular medical monitoring	1.30 (0.78-2.16)	0.299	-	-
Therapeutic modification	2.14 (1.41-3.26)	≤ 0.001	1.98 (1.11-3.53)	0.020
Sedentary life>	1.39 (0.94-2.05)	0.090	-	-
Smoking	0.63 (0.33-1.18)	0.150	-	-
Body Mass Index (BMI)	1.05 (1.02-1.09)	0.002	1.05 (1.00-1.10)	0.019
Dyslipidemia	2.16 (1.45-3.20)	≤ 0.001	2.07 (1.28-3.33)	0.003
**Paraclinical findings**				
HbA1C	0.94 (0.86-1.02)	0.164	-	-
FBG	0.86 (0.66-1.12)	0.289	-	-
Total cholesterol	1.18 (0.71-1.95)	0.510	-	-
Triglycerides	1.22 (0.91-1.64)	0.179	-	-
HDL-C	0.63 (0.20-1.98)	0.433	-	-
LDL-C	1.15 (0.55-2.41)	0.699	-	-

OHA: oral hypoglycemic agents; FBG: fasting blood glucose; HDL: high-density lipoprotein; LDL: low-density lipoprotein

In the multivariable analysis, age 50-59 years (OR = 2.00, 95% CI: 1.10-3.64, p = 0.023), age ≥60 years (OR = 4.74, 95% CI: 2.47-9.10, p ≤ 0.001), diabetes duration of 5-10 years (OR = 2.13, 95% CI: 1.17-3.86, p = 0.013) or >10 years (OR = 2.28, 95% CI: 1.14-4.53, p = 0.018), therapeutic modifications (OR = 1.98, 95% CI: 1.11-3.53, p = 0.020), BMI (OR = 1.05, 95% CI: 1.00-1.10, p = 0.019), and hypolipidemia treatment (OR = 2.07, 95% CI: 1.28-3.33, p = 0.003) were identified as independent risk factors for hypertension in T2DM patients (Table 3).

## Discussion

Hypertension is a highly prevalent disease among individuals with diabetes, the prevalence being more than twice as high in individuals with diabetes compared with those without diabetes. This study aimed to assess the prevalence and risk factors associated with hypertension among patients with type 2 diabetes mellitus (T2DM). Previous studies have shown that hypertension in patients with diabetes has been associated with an increased rate of microvascular (retinopathy, nephropathy, and neuropathy) and macrovascular (atherosclerosis) complications. Indeed, macrovascular complications are responsible for the majority of deaths in patients with T2DM [20]. Our findings revealed a high prevalence of hypertension (70.2%) in the study population. Several factors were independently associated with hypertension, including older age, longer duration of diabetes, therapeutic modifications, dyslipidemia, and higher BMI levels.

The results of our study revealed that hypertension is widespread in our population, with a prevalence of 70.2%. These findings are confirmed by the results of the study on the high prevalence of hypertension (70.4%) in patients with T2DM in three Moroccan regions, namely Fez, Salé, and Taounate [21]. These results of hypertension confirm that this problem has become a serious healthcare issue in our country. However, the current results are greater than the reported prevalence in other Arab populations (22% in Tunisia, 38% in Bahrain, 44% in Oman, 53% in the Kingdom of Saudi Arabia, 64.5% in Qatar, and 68% in Egypt) [22-27]. In comparison with other populations, this prevalence is less than the prevalence in the UK African-Caribbean population (82%) and much greater than the percentages reported among patients with diabetes in Turkish (32%), Taiwanese (39%), and Gaborone (61.2%) populations [28-31].

In this study, we found that the risk of developing hypertension in patients with T2DM is increased by a factor of 2.00 in patients aged 50-59 years and by a factor of 4.74 in patients aged 60 years or older. This association is consistent with the research findings and results of other studies [21,32]. This association could be due to age-associated changes in arterial stiffening and thickening [33]. The intima media thickening induced by aging affects the integrity of the endothelium and diminishes the availability of vasodilators like nitric oxide [34]. Indeed, the rigidity of the arterial walls perturbs the normal blood circulation, providing favorable conditions for the accumulation of calcium and fatty deposits inside the arteries, thus causing further narrowing of the arteries and hypertension [34-36].

The results of our study further showed that the risk of the development of hypertension increased among patients with T2DM for 5 to 10 years or more than 10 years. In this regard, various studies have identified the duration of diabetes as a risk factor not only for hypertension but also for other macro- and microvascular complications of T2DM [37-39].

The current study showed that having an elevated BMI was a significant risk factor for developing hypertension in our population, which confirms the results of similar studies conducted in other regions of Morocco and other countries [21,40,41]. Therefore, obesity has become one of the modifiable risk factors strongly associated with hypertension in patients with type 2 diabetes due to its role in insulin resistance [42]. Thus, the findings of our study strengthen the necessity of focusing on increasing the physical activity level of people with T2DM in Morocco.

Moreover, patients with T2DM on lipid-lowering therapy have a 2.07 times greater risk of developing hypertension. This association can be explained by many reasons. Firstly, dyslipidemia could alter the function of the endothelium, perturbing the balance between relaxation and contraction factors derived from the endothelium and diminishing the production of nitric oxide, resulting in endothelial cell dysfunction and disturbances in blood pressure regulation [43,44]. Furthermore, the endothelial lesions would cause a reduction in vasomotor activity and a deregulation of vasoconstriction in patients with dyslipidemia, provoking a further increase in blood pressure [45]. Another explanation for this association involves the renin-angiotensin-aldosterone system. Indeed, angiotensin II increases the risk of hypertension and atherosclerosis by stimulating angiotensin type 1 receptors, increasing lipid uptake into cells, production of free radicals, and vasoconstriction [46].

Our findings have significant clinical and public health implications. The high prevalence of hypertension among patients with T2DM underscores the urgent need for early detection, regular monitoring, and effective management strategies [15]. Given older age, duration of diabetes, higher BMI, dyslipidemia, and therapeutic modifications were identified as key risk factors, targeted interventions such as lifestyle modifications (weight control, physical activity), lipid management, and individualized treatment adjustments should be prioritized [7]. From a public health perspective, these results indicate the necessity of integrating hypertension screening into routine diabetes care in Morocco. Strengthening primary healthcare systems to improve patient education, antihypertensive therapy, and multidisciplinary care approaches could help reduce cardiovascular risks associated with T2DM [21].

This study provides valuable information on the prevalence and risk factors of hypertension among patients with T2DM. One of its strengths is the relatively large sample size (503 patients), allowing for a robust statistical analysis of associations between hypertension and various risk factors. However, certain limitations should be acknowledged. First, a cross-sectional design prevents any causal inference between risk factors and hypertension. Second, the study was conducted in a single region, which may limit the generalizability of the findings to all Moroccan diabetic populations, particularly those in rural areas. Third, some confounding factors, such as dietary habits and stress levels, were not assessed, which could have influenced the observed associations. Future research should include longitudinal designs and diverse geographic representation to strengthen the understanding of hypertension in this population.

## Conclusion

This study found a high prevalence of hypertension (70.2%) among patients with T2DM. Independent risk factors included older age, longer diabetes duration, higher BMI, dyslipidemia, and therapeutic modifications. These findings emphasize the importance of early hypertension screening and continuous monitoring in diabetic patients to prevent cardiovascular complications. Given the strong associations identified, integrating lifestyle modifications and optimizing treatment plans may help improve blood pressure control in this population. However, due to the cross-sectional design, causal relationships cannot be established. Further longitudinal studies are needed to assess the long-term impact of these risk factors.

### What is known about this topic

- Hypertension is highly prevalent among patients with T2DM and significantly increases the risk of cardiovascular complications;
- Several factors, including age, obesity, and dyslipidemia, have been associated with hypertension in diabetic populations worldwide;
- Data on the prevalence and risk factors of hypertension in North African diabetic populations, including Morocco, remain limited and underreported.

### What this study adds

- This study reports a 70.2% prevalence of hypertension in Moroccan T2DM patients, highlighting a major public health concern;
- Older age, longer diabetes duration, higher BMI, dyslipidemia, and therapeutic modifications are key independent risk factors for hypertension in this population;
- Findings emphasize the need for early screening and tailored interventions to manage hypertension in Moroccan diabetic patients.
